# Risk of Human Papillomavirus (HPV) Infection and Cervical Neoplasia after Pregnancy

**DOI:** 10.1186/s12884-015-0675-0

**Published:** 2015-10-07

**Authors:** Helen Trottier, Marie-Hélène Mayrand, Maria Luiza Baggio, Lenice Galan, Alex Ferenczy, Luisa L. Villa, Eduardo L. Franco, for the Ludwig-McGill Cohort Study Group

**Affiliations:** Division of Cancer Epidemiology, Department of Oncology, McGill University, Montreal, Canada; Department of Social and Preventive Medicine, Université de Montréal, Montreal, Canada; Sainte-Justine Hospital Research Center, Department of Social and Preventive Medicine, Université de Montréal, 3175 Côte Sainte-Catherine, Room A-830, Montreal, QC H3T 1C5 Canada; Department of Obstetrics and Gynecology, Université de Montréal and CRCHUM, Montreal, Canada; Ludwig Institute for Cancer Research, São Paulo, Brazil; Department of Pathology, McGill University and Jewish General Hospital, Montreal, Canada; Department of Radiology and Oncology, School of Medicine, University of São Paulo, São Paulo, Brazil

**Keywords:** Cervical neoplasia, Pregnancy, Human papillomaviruses (HPV)

## Abstract

**Background:**

Parity is well established as a risk factor for cervical cancer. It is not clear, however, how pregnancy influences the natural history of HPV infection and cervical neoplasia. Our objective was to study the risk of HPV infection and cervical squamous intraepithelial lesions (SIL) after pregnancy.

**Methods:**

We used the Ludwig-McGill cohort study which includes 2462 women recruited in Sao Paulo, Brazil in 1993–97 and followed for up to 10 years. Cellular specimens were collected every 4–6 months for Pap cytology and HPV detection and genotyping by a polymerase chain reaction protocol. Study nurses recorded pregnancy occurrence during follow-up. HPV and Pap results from pregnant women were available before and after, but not during pregnancy. The associations between pregnancy and post-partum HPV infection/SIL were studied using generalized estimating equation models with logistic link. Adjusted odds ratios (OR) were estimated with empirical adjustment for confounding.

**Results:**

We recorded 122 women with a history of pregnancy during follow-up. Of these, 29 reintegrated the cohort study after delivery. No association between HPV and pregnancy was found. A single SIL case (high grade SIL) occurred post-partum. Likewise, there was no association between pregnancy and risk of low grade SIL or any-grade SIL at the next visit (adjusted OR = 0.84, 95 % CI: 0.46-15.33) after controlling for confounders.

**Conclusions:**

No associations were found between pregnancy and HPV or LSIL. The single observed case of HSIL post-partum was more than would be expected based on the rate of these abnormalities among non-pregnant women. As this association was found with only one case, caution is required in the interpretation of these results.

## Background

The central causal role of the so-called high oncogenic–risk (HR)-human papillomavirus (HPV) genotypes, such as HPV-16 in cervical carcinogenesis, has been established [1]. HPV is a necessary but not sufficient cause of virtually all cases of cervical cancer worldwide. HR-HPV infection also causes a substantial proportion of other anogenital neoplasms and oral squamous cell carcinomas [2]. HPV infections are one of the most common sexually transmitted conditions in the world although the vast majority are transient, with only a small proportion becoming persistent and leading the development of cervical cancer [3]. The fact that HPV infection does not always progress to neoplastic disease suggests that interpersonal variations in the immune system may play a role in the clearance of HPV infections and/or in their acquisition. Other viral or environmental factors may also play a role [4]. For example, parity is a well-established risk factor for cervical cancer [5]. It is not clear, however, exactly how pregnancy influences the natural history of human papillomavirus (HPV) infection and cervical neoplasia. To our knowledge, there is no prospective study of the risk conferred by pregnancy on both HPV infection and cervical intraepithelial neoplasia. The aim of this prospective study was to analyse the risk of HPV infection and cervical squamous intraepithelial lesion following pregnancy.

## Method

### Subject recruitment

The women included in this study were enrolled into the Ludwig-McGill cohort, a longitudinal investigation of the natural history of HPV infection and precursor lesions of cervical cancer. A detailed description of the design and methods of the study has been published previously [6]. Briefly, women attending a maternal and child health program catering to a low-income neighborhood in São Paulo (Brazil) were recruited between 1993 and 1997 and followed for up to 10 years. Women were eligible to participate if they: 1) were between 18 and 60 years of age, 2) were permanent residents of São Paulo, 3) were not currently pregnant and had no intention of becoming pregnant during the first year of follow-up, 4) had an intact uterus and no current referral for hysterectomy, 5) reported no use of vaginal medication in the previous 2 days and 6) had no reported treatment for cervical disease in the previous 6 months. Subjects gave a signed informed consent. The study protocol was approved by institutional ethical and research review boards of the participating institutions in Canada (McGill University Research and Ethics Board, Montreal) and in Brazil (Ludwig Institute for Cancer Research São Paulo Branch Institutional Review Board (IRB) and by the IRB of the Hospital e Maternidade Escola Vila Nova Cachoerinha Dr Mario Altenfender, São Paulo).

The study enrolled 2528 women, corresponding to a 70 % response rate and subsequently, 66 ineligible women were excluded. Follow-up for the remaining 2462 women consisted of 1 visit every 4 months for the first year and 2 visits per year thereafter. Cervical specimens were taken for Papanicolaou (Pap) cytology and HPV testing at every visit with a fixed-sampling area Accelon device. For the first 4 visits and for each annual visit thereafter, subjects answered a nurse-administered questionnaire designed to collect information on socio-demographic, lifestyle, sexual, reproductive and contraceptive characteristics. Pregnancy status was registered by study nurses along with date and procedures of visits, reasons of dropouts and reintegration, and other study-relevant incidents. Pregnant women were excluded from the study during pregnancy and offered to be reintegrated in the study after a minimum of 45 days following delivery, if there was no clinical impediment and if they consented. HPV and Pap results from pregnant women were available before and after, but not during pregnancy.

### Cervical cells specimen

An Accelon biosampler (Medscand, Inc., Hollywood, Florida) was used to collect ecto- and endocervical samples for each visit and a Pap smear was prepared on a glass slide and fixed with 95 % ethanol. The sampler containing the residual exfoliated cells was immersed in a tube containing Tris-EDTA buffer (pH 7.4) and agitated to release the cells. The tubes containing cell suspensions were frozen until testing. Samples were then sent to the Ludwig Institute for storage and testing. Pap smears were sent to the study centre in Canada for cytology reading by one of the co-authors (AF). Cytopathology reports were based on the 1991 Bethesda system for cytological diagnoses [7]; the Pap smears were read ‘blinded’ to all other test results for the same sample and for the same woman and findings were classified as normal, atypical squamous cells of undetermined significance (ASCUS), atypical glandular cells of undetermined significance (AGUS), low grade squamous intraepithelial lesion (LSIL), or high grade squamous intraepithelial lesion (HSIL).

### HPV DNA testing

The Accelon device containing ecto- and endocervical cells was placed in a tube containing Tris-EDTA buffer (pH 7.4). DNA was extracted, purified by spin column chromatography, and amplified by polymerase chain reaction (PCR), using the MY09/11 and PGMY protocols [8, 9] for detection of HPV DNA. Typing of amplified products was performed by dot-blot hybridization with individual oligonucleotide probes and by restriction fragment length polymorphism (RFLP) analysis. This method identified more than 40 HPV genital types. Amplified products that hybridized only with a generic probe and were unidentifiable in RFLP analysis were classified as positive for unknown types. The genotypes tested included high oncogenic risk (HR-) HPV types 16, 18, 31, 33, 35, 39, 45, 51, 52, 56, 58, 59, 66, 68, 73 and 82, and low oncogenic risk (LR-) HPV types 6, 11, 26, 32, 34, 40, 42, 44, 53, 54, 57, 61, 62, 67, 69, 70, 71, 72, 81, 83, 84 and 89 (unknown types considered LR-HPVs) [10, 11]. We included more than 30 type-specific positive controls in hybridization membranes to control for experimental variation between different membranes. DNA specimen quality was checked by amplification of a 268-bp human β–globin gene region [8]. Specimens were tested blindly and precautions were taken to prevent contamination. Samples that were negative for both HPV and β–globin were considered inadequate for analysis.

### Statistical analysis

We explored the relationships between pregnancy and outcomes such as HPV infection and cervical squamous intraepithelial lesions (SIL) using generalized estimating equation (GEE) via a logistic regression link, which take into account the clustering within each individual caused by the repeated-measurements design. GEE models were based on an exchangeable correlation pattern. We studied the association between pregnancy and the presence of HPV, LSIL and HSIL. Post-partum visits were compared to other usual visits for the presence of HPV or SIL. We explored the relationship between HPV infection and pregnancy by considering grouped HPV genotypes per their oncogenic potential (high-risk and low-risk as described above), per their phylogenetic relationship within the genus Alpha-papillomavirus (species 3, 5, 6, 7, 9, 10) [12] and grouped HPV per their general tropism for the cervix or the vagina [13]. HPV group 1 (benign) included HPV types 6, 11, 32, 40, 42, 44 (cervical and vaginal species 1, 8, 10); HPV group 2 included HPV types 16, 18, 26, 31, 33, 35, 39, 45, 51, 52, 53, 56, 58, 59, 66, 67, 68, 69, 70, 82 (cervical species 5, 6, 7, 9, 11); and HPV group 3 included HPV types 57, 61, 71, 72, 62, 81, 83, 84, 89 (vaginal species 3, 4, 15).

We estimated crude and adjusted odd ratios (ORs) with respective 95 % confidence intervals (CI). For adjusted estimates, we controlled for empirical confounders using a 5 % change-in-estimate method (variables that changed the estimates by +/− 5 % were included in the model) considering the following variables: age at enrolment (linear), race (white, non-white), marital status (single, married, widowed, separated, unmarried but living with partner), income (quartiles of income), smoking (never, current, former), age at first sexual intercourse (≤15, 16–17, 18–19, 20+), number of previous pregnancies (0–1, 2–3, 4–6, 7+), Pap testing before enrollment in the cohort (yes, no) and lifetime number of sexual partners (0–1, 2–3, 4+). For the analysis concerning the association between SIL and pregnancy, we also considered HPV status (negative, LR-HPV only, any HR-HPV) as a potential confounder or mediator.

## Results

A total of 2475 women were included and provided altogether 24,558 visits. The mean and median follow-up time were 5.1 (SD = 3.2 years) and 6.4 (inter-quartile range = 1.0-7.5) years, respectively. The mean age at enrolment was 32.7 years (SD = 8.8; median = 32, range: 18–59) and most women were white (64 %). We recorded 122 women with a history of pregnancy during follow-up. After giving birth, 29 (23.8 %) women reentered the study. The mean time between visits for reintegration into the cohort was 3.9 years (SD = 1.6).

Table 1 describes the characteristics at baseline (enrolment) according to the pregnancy status in the course of follow-up. Women who became pregnant over the course of follow-up were younger than those in the remainder of the cohort. The distribution of other age-dependent variables also differed according to pregnancy outcomes, e.g., women who did not become pregnant were more likely to have a normal cytology result and to be HPV negative at baseline. Table 1 also provides baseline data for women who had a pregnancy but who did not reintegrated the cohort after pregnancy. Overall, women lost to follow-up after pregnancies were largely comparable to those who reintegrated into the cohort after giving birth.

**Table 1:** Characteristics at baseline (at enrolment in the cohort) according to pregnancy status in the course of follow-up

Characteristics at baseline		Women without pregnancy in the course of follow-up	Pregnant women who reintegrated the cohort following pregnancy	Pregnant women who did not reintegrate the cohort following pregnancy	*p-value
		*N* = 2353	*N* = 29	*N* = 93	
Age (mean, SD)		32.7 (8.8)	26.7 (6.1)	27.2 (5.4)	0.609
Age (median, IQR)		32.0 (26.0-39.0)	26.0 (21.0-31.5)	27.0 (22.3-31.0)	
		(%)	(%)	(%)	
Cytology	Negative	96.2	86.2	90.0	
	ASC/AGUS	1.7	0.0	5.0	0.221
	LSIL	1.2	6.9	1.3	
	HSIL	0.7	6.9	3.8	
HPV status	Neg	82.6	72.4	77.5	
	LR-HPV only	6.4	10.3	5.0	0.600
	Any-HR	10.1	17.2	16.3	
Ethnicity	White	64.9	55.2	53.8	
	Nonwhite	35.1	44.8	46.3	0.895
Marital status	Single	10.3	6.9	10.0	0.148
	Married	48.3	48.3	35.0	
	Unmarried, but living with partner	33.1	37.9	53.8	
	Widowed/Separated	8.3	3.5	1.3	
Quartiles of income	1 (lowest)	24.8	37.9	27.9	
	2	24.8	17.2	25.3	0.069
	3	25.3	34.5	17.7	
	4 (highest)	25.1	10.3	29.1	
Age at first intercourse	20+	26.4	13.8	12.5	
	18-19	21.2	0.0	25.0	0.324
	16-17	25.6	37.9	26.3	
	<=15	26.9	48.3	36.3	
Previous pregnancy	0-1	16.9	24.1	20.0	
	2--3	42.8	24.1	42.5	
	4--6	30.1	44.8	27.5	0.231
	7+	10.2	6.9	10.0	
Lifetime number of sexual partners	0-1	44.3	44.8	41.3	
	2-3	34.7	31.0	40.0	
	4+	20.9	24.1	18.8	0.662
Ever had PAP cytology before	No	5.0	3.5	12.5	
	Yes	95.0	96.6	87.5	0.166
Smoking	Never	47.6	51.7	40.0	
	Current	34.9	27.6	43.8	0.312
	Former	17.4	20.7	16.3	

*P-value to test the difference between pregnant women who reintegrated and those who did not reintegrate the cohort following pregnancy. P-values estimated with Pearson's chi-squared for categorical variables and student t-test for continuous variables

Total may not sum 100 % due to missing data. *SD* standard deviation, *IQR* interquartile range

Table 2 presents the crude and adjusted ORs for the association between pregnancy and post-partum HPV infection and SILs. Six of the post-pregnancy visits were HPV positive (20.7 %), whereas 3787 out of 23,735 (16.0 %) visits without an intervening pregnancy yielded the same finding. We found no evidence that pregnancy increased the risk of detecting HPV at the next visit post-partum irrespective of the categories used to group HPV genotypes (range of adjusted ORs: 0.80-1.86; none statistically significant). Table 2 also shows the association between pregnancy and cytological abnormalities. A single SIL case (an HSIL) occurred post-partum. Likewise, there was no association between pregnancy and LSIL or any-grade SIL at the next visit (adjusted OR = 0.84, 95 % CI: 0.46-15.33) after controlling for confounders. The single observed case of HSIL post-partum was more than would be expected based on the rate of these abnormalities among non-pregnant women even after accounting for multiple confounders (OR = 8.75, 95 % CI: 1.00-77.03).

**Table 2:** Crude and adjusted odds ratios (ORs) for the association between HPV detection or squamous intraepithelial lesion (SIL) and pregnancy

Outcome	Post pregnancies observation visits (n)		Crude OR	95 % CI	Adjusted OR^c^	95 % CI
	No pregnancy	Pregnancy				
HPV^b^	^a^HPV (−/+)	^a^HPV (−/+)				
Any HPV	19948/3787	23/6	0.85	(0.33–2.14)	0.80	(0.31–2.07)
Any HR-HPV	19948/2281	23/3	0.80	(0.24–2.65)	0.80	(0.24–2.71)
LR-HPV only	19948/1506	23/3	1.15	(0.31–4.23)	1.18	(0.34–4.15)
Species^b^						
3	19948/638	23/1	1.49	(0.26–8.50)	1.32	(0.22–7.97)
5	19948/343	23/1	2.32	(0.35–15.23)	1.86	(0.28–12.15)
6	19948/505	23/1	1.06	(0.11–9.83)	0.91	(0.10–8.12)
7	19948/645	23/1	1.23	(0.20–7.60)	0.98	(0.15–6.24)
9	19948/1407	23/1	0.48	(0.07–3.21)	0.43	(0.07–2.59)
10	19948/303	23/1	1.26	(0.09–17.40)	1.29	(0.13–12.93)
Group						
1 (species-1/8/10/13)	19948/399	23/1	0.93	(0.64–13.53)	1.05	(0.10–10.53)
2 (species-5/6/7/9/11)	19948/2566	23/4	0.87	(0.29–2.56)	0.77	(0.27–2.21)
3 (species-2/3/4/15)	19948/716	23/1	1.41	(0.26–7.64)	1.25	(0.21–7.29)
Squamous intraepithelial lesions (SILs)	SIL (−/+)	SIL (−/+)				
Low-grade SILs	23845/258	28/0	--	--	--	--
High-grade SILs	23845/76	28/1	7.77	(0.93–65.08)	8.75	(1.00–77.03)
Any-grade SILs	23845/334	28/1	0.91	(0.05–18.27)	0.84	(0.46–15.33)

^a^Excludes women-visits for which HPV testing was invalid or missing

^b^See text for definition of grouped HPV genotypes

^c^Adjustment for empirical confounders was done using a 5 % Change-in-Estimate Method (variables that changed the estimates by +/− 5 % were included in the model) considering the following variables as a potential confounder: age at enrolment (linear), race (white, non-White), marital status (single, married, widowed, separated, unmarried but living with partner), income (quartiles of income), smoking (never, current, former), age at first sexual intercourse (≤15, 16–17, 18–19, 20+), number of previous pregnancies (0–1, 2–3, 4–6, 7+), Pap testing before enrollment in the cohort (yes, no), and lifetime number of sexual partners (0–1, 2–3, 4+). Adjustment for HPV status (negative, LR-HPV only, any HR-HPV) was considered for the analysis of SILs. Confounding variables added to the multivariate models for HPVs (variables that changed the OR for the relation between pregnancy and HPV status by +/− 5 %) were: number of previous pregnancies, age at first sexual intercourse, age, race and smoking status. For SILs, confounding variables added to the multivariate models were: HPV status, age, smoking, race, age at first sexual intercourse, number of previous pregnancies and Pap testing before entering the cohort

## Discussion

In this longitudinal study, we analysed the risk of HPV infection and cytological abnormality following pregnancy. No association was detected between pregnancy and LSIL, nor for post-partum HPV infection. Although, the single observed case of HSIL post-partum was more than would be expected based on the rate of these abnormalities among non-pregnant women, this association was found with only one case, which calls for caution in interpreting these findings.

There are plausible mechanisms whereby pregnancy may influence the natural history of HPV infection and progression to clinical lesions. Increased levels of estrogen or growth hormone associated with pregnancy such as human chorionic gonadotropin (hCG) may increase HPV molecular activity [14, 15]. Studies on condyloma acuminata and laryngeal recurrent respiratory papillomatosis have shown that these lesions increase in number and size during pregnancy [16]. Others suggested that a reduced humoral response to HPV may occur in pregnant women [17].

Our finding of no increased risk of HPV detection with pregnancy concurs with other reports [18–27]. Only a few showed a higher risk of HPV infection in pregnant women [28–30]. The higher risk of HPV infection in these studies could be explained by confounding variables related to sexual activity, as pregnancy and HPV infection are both acquired through sex. Some studies have shown a higher risk of HSIL with pregnancy. Morimura et al. (2002) [31] have shown that the proportion of women with of abnormal Pap test results was significantly higher during pregnancy. Jensen and colleagues (2013) [32] found that childbirth increased the risk of HSIL, over and above the risk of persistent HPV infection. Taken together, those studies suggest, that careful monitoring of HPV positive women in the post-partum period may be warranted.

It is not clear however, whether or not the risk of progression of HPV infection to cervical neoplasia (preinvasive and invasive cancer) is higher when detected during pregnancy. Studies on the progression of biopsy proven cervical neoplasia during pregnancy are rare, since biopsies tend to be avoided during pregnancy. When Pap results are used to estimate risk, reports provide a wide range of estimates: 10–70 % of cytological abnormalities found during pregnancy regress, 25–89 % persist without progression and 3–30 % progress [33–46]. Studies on evolution of HSIL during pregnancy and the post-partum period show a range of progression to micro-invasive carcinoma from 0 to 14.2 % and a very low risk of progression to frankly invasive cancer (0.1 %) (reviewed in Serati et al., [44]).

This study has limitations. It is possible that residual confounding may explain our findings. For example, lack of control for women’s partners’ sexual behaviour could create confounding if pregnancy is associated with a higher likelihood of partners’ extra-conjugal sexual relationships. However, this potential confounding bias is unlikely to have occurred, as no association was found for HPV infection or LSIL. We also used cytology and not histology results in this analysis. However, Morimura et al. (2002) [31] has shown that the accuracy of cytology Pap testing is similar between pregnant and non-pregnant women. As this potential information bias is non differential, it would have only underestimated the relative risk we found. Finally, most of the women in our cohort already had their children at the time of recruitment and only a small number of women experienced a pregnancy within the cohort. Moreover, a criterion for recruitment in our cohort was not being currently pregnant and no intention of becoming pregnant during the first year of follow-up, which reduced the number of pregnancies in the cohort and the number of women who reentered the cohort after birth.

One of the strengths of our study was the use of a longitudinal design approach including prospective collection of data before and after pregnancy. This limits the potential for bias that would have occurred if pregnant women were more likely to be screened compared to non-pregnant women because all pregnant women in our study were similarly followed-up in the cohort prior to becoming pregnant. We also applied a conservative control for confounding including sexual behaviour and variables that were different between pregnant and non pregnant women such as age.

## Conclusions

In conclusion, we found no association between pregnancy and HPV infection and cervical lesions but admittedly, our cohort included a limited number of pregnant women. A single SIL case (an HSIL) occurred post-partum which calls for caution in interpretation.
